# The Eighth Periodical International Ophthalmological Congress

**Published:** 1895-03

**Authors:** Alvin A. Hubbell

**Affiliations:** Buffalo, N. Y.


### Reports of Societies.

### THE EIGHTH PERIODICAL INTERNATIONAL OPHTHALMOLOGICAL CONGRESS, HELD AT EDINBURGH, AUGUST 7–11, 1894.

By ALVIN A. HUBBELL, M. D., Buffalo, N. Y.

(*Concluded from page 411.*)

Priestly Smith (3) had done the scleral puncture as an adjunct to iridectomy in the treatment of glaucoma fifty times in noncongestive cases under cocaine anesthesia, in others under chloroform. A valvular puncture is made with a von Grafe's cataract knife introduced through the sclera at least five millimeters back of the corneal margin and then rotated and removed. A small, leaking puncture remains. No internal hemorrhage has ever occurred. After iridectomy it prevents the lens being pushed forward ; before iridectomy it has the same effect. It may be of use in some cases where iridectomy is inadvisable.

Abadie said that he had seen many glaucoma patients subjected to sclerotomy, but not one had ever been definitely cured, while iridectomy had preserved the sight of many. He accepted Priestly Smith's method of puncture as a means of facilitating iridectomy, but without attributing to it any curative value.

Parent had had recourse to puncture of the sclera several times and always with complete satisfaction.

Pflüger had for several years used posterior sclerotomy in severe cases of glaucoma as a preliminary to iridectomy, and his experiences coincided precisely with those of Priestly Smith.

McHardy, of London, had found the iridectomy complicated and not facilitated, immediately after the scleral puncture from the marked softening that the latter produced. He made very large iridectomies in glaucoma and in the malignant form he obtained successful results by extracting the lens.

Power had performed an operation which he said was almost identical with that of Priestly Smith and which was originally proposed by Mr. Hancock, of the Royal Westminister Ophthalmic Hospital. It was done with a Beer's knife, which was passed into the vitreus through the sclera three or four millimeters from the margin of the cornea. It is in acute glaucoma that it is most successful. He had almost given up operating in chronic glaucoma.

Menacho had practised the old posterior sclerotomy and was satisfied that it was efficacious in relieving the acute symptoms of glaucoma.

Priestly Smith concluded the discussion by reminding his hearers that his paper referred to scleral puncture as an *adjunct to iridectomy* and not as an independent operation. The withdrawal of a small quantity of fluid from the vitreous chamber does not, cannot, in fact, immediately alter the depth of the anterior chamber.

Mules (6 *a*) had performed a new operation for ptosis, in which a small elliptical piece of cartilage is removed and, by wires carried to half an inch above the eyebrow, the lid is drawn up to the required degree.

Little had seen the good results in a case operated on by Mules.

Pflüger (7) recommended drainage of the anterior chamber in infections of it, as well as in keratoconus and in some cases of glaucoma and detachment of the retina. He uses a small piece of guttapercha in the form of a cross, the short part being introduced through an opening in the cornea to the depth of about two millimeters into the anterior chamber. The other parts are fixed under the conjunctiva. If injections into the anterior chamber are necessary, this drain must be frequently changed. In conical cornea it may remain several days and its use should be often repeated.

Walker (8) had devised the following operation for relieving tension in chronic glaucoma: Dissect a small flap of conjunctiva about three to six millimeters close to the edge of the cornea; then thrust a small knife, with the base toward the iris, into the anterior chamber at the margin of the cornea and into this opening push the conjunctival flap.

The epithelial surface of the conjunctiva not uniting with the cornea, a permanent fistula and continuous drainage are established.

Landolt (28), after twenty years' attention to strabismus, had come to the conclusion that, in its correction, muscular advancement was better than tenotomy. After tenotomy the excursions of the eye are often limited, the caruncle often becomes retracted, the power of the operated muscle weakened and the eye sometimes has the appearance of being more prominent than the other. On the other hand, advancement gives results as good and complete without any of the drawbacks of a tenotomy and it is without danger. For example, there is no risk of over-correction, no insufficiencies, no retraction of the caruncle and no malposition of the eyeball.

In convergent strabismus he preferred to simultaneously advance both external recti muscles and follow the operation by orthoptic exercises. In muscular insufficiencies or slight strabismus a unilateral advancement would be sufficient. In divergent strabismus a tenotomy of the external rectus gives only a metric-angle of correction, while a complete correction may be obtained by advancement of the internal rectus.

It is only in high degress of strabismus that he combined a tenotomy of the antagonistic muscle with an advancement, and then the tenotomy is done sub-conjunctivally and without further detachment from the surrounding tissues.

Swanzy did not think that simple tenotomy should be discarded in favor of advancement to the extent recommended by Landolt. He believed the rules of von Graefe still hold good. He never used advancement alone, but frequently combined tenotomy with it in extreme cases. He found difficulty in deciding how much of the tendon should be removed to produce the desired effect. He also found that advancement sometimes causes disfigurement by reason of an unpleasant red lump being produced at the point of the new insertion. Does Landolt have any difficulty in securing a flat cicatrix ?

Pannas was of the same opinion as Landolt in regard to the superiority of advancement, especially in cases due to paralytic insufficiency. In concomitant strabismus, on the other hand, in which the equilibrium is not disturbed by paralysis or retraction, but by insufficient convergence, tenotomy is more certain. Advocates of advancement admit that it frequently fails to afford relief in the divergent strabismus of myopia. For these reasons he employs advancement merely as an adjuvant to tenotomy in marked divergent and in acute paralytic strabismus.

Roosa had used the method of advancement as a substitute for, or as an adjuvant to, tenotomy in very high degrees of convergent strabismus, or when one or more tenotomies had been performed. But to substitute advancement for tenotomy on account of the danger of diplopia, was to make a change from a slight to a more serious operation. Advancement should not be used as a general substitute for tenotomy. All refractive errors should be corrected.

Gruening, of New York, endeavored to avoid the more serious operation of advancement and obtained very satisfactory results, even in divergent strabismus, by the simple operation of tenotomy.

Stevens said that equality of rotation in all directions must be maintained in order to obtain the best results in strabismus operations and the perfect union of images in all positions. To maintain such equality, relaxation of corresponding muscles must be made. Tenotomy of one muscle and shortening of its opponent of the same eye is inconsistent with this principle. He believed that in most cases of strabismus the defect may be relieved and binocular vision obtained with perfect rotation in all directions by carefully graduated tenotomies on both eyes to be repeated after some months. But in every case the various forces are to be regarded.

Landolt replied that the criticisms of his esteemed colleagues proved that they do not examine, treat, operate or follow up their patients as he had done. Not one, for instance, had mentioned the measurements of the excursions of the eyes, of convergence, or of divergence, before or after operation. Yet this should be done. He agreed that great stress should be laid upon the correction of optical defects in the cure of strabismus. Operation is not sufficient to do away with a squint, but is only a step in a long and carefully directed orthoptic line.

Thier (31) had operated on thirty-eight cases of high myopia by discission and absorption of the crystalline lens. He passes the knife through the whole thickness of the lens and the posterior capsule. Rapid swelling of the lens is desired, and if cloudiness and swelling do not develop within eight or ten days he repeats the discission. Young patients are most suitable, but he had operated in one case successfully at forty-eight years of age.

He had operated on thirty-eight patients. In eleven he had operated on both eyes, and in sixteen on one eye. In nearly all a marked improvement of vision had been produced.

Fukala (32), in speaking on this same subject, said that he operated by discission and followed this by paracentesis and evacuation of the lens. He operated on all suitable cases under forty years of age, avoiding those affected with chronic choroiditis or detachment of the retina. He was opposed to the immediate extraction of a transparent lens, as he thought it liable to be followed by a dangerous hyperemia of the choroid.

Schmidt-Rimpler (Göttingen) considered it necessary to watch the patients for a long time after one of these operations. In one case, operated by discission on one eye, the myopia had progressed in the same manner in the aphakial eye as in the other, with the development of the same choroidal lesions. From this he concluded that, in young persons, the double operation was preferable.

Pflüger had operated on forty cases with good results. He differed with Thier and Fukala in believing that choroidal changes are not a contra-indication. There are few cases of high myopia without them. He does not dare to divide the lens as deeply as does Thier, for the vitreous humor might pass into the anterior chamber and out through the corneal wound. The tension should be watched after discission, and if it occurs it may be controlled by repeated paracentesis.

Thier, in reply, said that with good antisepsis he had little fear of the presentation of the vitreous outside, and the large discission hastened the termination of the treatment and left an unobstructed pupil. Furthermore, the contraction of the capsule, which might otherwise occur and cause irritation, is prevented.

Fukala said that, as a contra-indication to the operation, he referred to those choroidal changes which affected the macula and those which caused numerous exudates into the vitreus.

Noyes (33) described an operation which he had performed in a case of closure of the pupil from irido-cyclitis after cataract extraction. With a von Graefe's cataract knife he makes a section on each side of the cornea, and before withdrawing the knife plunges the point into the iris near the center. If there is hemorrhage he injects into the anterior chamber saline water by one of these openings and out by the other. When the hemorrhage ceases, he incises the iris with de Wecker's scissors, on both sides. The hemorrhage is again treated by saline injections into the anterior chamber, as before. Finally, the flap of iris is drawn out, on either side, by a hook and cut off.

Darier referred to a similar operation which had been done by Abadie, in which the iris was incised in the form of a V and the iris flap drawn out from the opposite side and cut off.

McHardy (41) reported the results of treatment of immature cataract by preliminary iridectomy with trituration of the lens. He had had three different series:

| | 1st Series. | 2d Series. | 3d Series. |
|---|---|---|---|
| Number of cases | 25 | 100 | 49 |
| Unduly early extraction required | 4 per cent. | 2 per cent. | 4 per cent. |
| Second and third trituration required | 28 " " | 9 " " | 4 " " |
| Troublesome, but controlable, iritis | 12 " " | 17 " " | 2 " " |
| Slight loss of vitreus during extraction | 28 " " | Not noted. | 4 " " |
| Total failure to restore sight | None. | 3 per cent. | None. |

Comparative observations were also made in cases in which a preliminary iridectomy had been done with and without trituration:

| Operation. | With Iridectomy. | Without Iridectomy. |
|---|---|---|
| Cases | 37 | 21 |
| Cortex semi-fluid, non-sticky | 80 per cent. | ——— |
| " sticky | None. | 33 per cent. |
| " hard | 20 per cent. | 66 " " |
| Cystoid cicatrix | 3 " " | 10 " " |
| Secondary capsulotomy | 21 " " | 35 " " |

He had been using this method of trituration of the lens for nine years. He obtained complete ripening in immature senile cataracts in from eight days to eight weeks, and the procedure was a safe one. He has the eye well under the influence of atropine at the time of the preliminary operation. After waiting six to eight weeks, he extracts with as much safety and as good results as in cases where the maturity was spontaneous, and it was free from the evil effects which follow the removal of immature cataracts.

Noyes believed there was a place for trituration of immature cataracts. He found the indication in cases of soft cataracts, with broad striæ, which, if operated on, would leave a large quantity of cortex behind to excite iritis and produce a bad secondary cataract. But this trituration and maturation can be perfectly well done without iridectomy. Within the past five years he had operated on cases of sclerosed lenses, regardless of the amount of transparency, and he had found that where sclerosis of the nucleus could be discerned, the cortex was as perfectly evacuated as if the whole lens were opaque. The incision included almost half of the cornea, no iridectomy was done, and, if some obscurity resulted, irrigation with a warm physiological solution of common salt cleared the pupil. The stream was directed by a rubber bulb, without introducing its point into the wound. He felt less anxiety, however, in a case in which the lens was fully opaque.

Hill Griffith, of Manchester, had employed trituration with advantage, and recalled some of his experience.

Theobold (43) advocated the use of large probes in the treatment of strictures of the nasal duct, and described his probes and his method of using them, all of which has long been familiar to American ophthalmologists.

Grüning had had excellent success with Theobold's probes, and confirmed Theobold's experience.

Roosa was not in favor of Theobold's method exclusively. Many cases are purely those of catarrh, and not of stenosis, and very little and simple treatment is often all that is necessary. He was well satisfied with his own results in employing Bowman's method, with such deviation as would suggest itself to an intelligent man.

Thompson, of Indianapolis, had treated such cases during the last twenty-three years by all sorts of methods. He began with large probes about fourteen years ago and had used them in every way, frequently and less often, and in some instances "large enough for a horse." Yet he has had patients return in less than a year, sometimes earlier, with the ducts just as small and the disease as bad as before treatment.

Risley, of Philadelphia, thought the treatment by large probes was opposed to the normal physiological process. An opening in the canaliculus sufficiently large to admit the largest of Theobold's series, destroyed the physiological relation and function of the parts. The lachrymal duct, in its normal condition, is a capillary tube and not a drain-pipe.

Chibret, in view of the fact that probing does not cure all cases of epiphora, had found much relief in diminishing the amount of secretion by ablation of the inferior lachrymal gland, according to the method of de Wecker. This reduced the secretion to one-fourth.

Priestly Smith favored wearing for long periods lead or silver probes, with the upper end bent so as to lie in the open canaliculus.

Lee, of Liverpool, recommended prolonged irrigation of the lachrymal passages, through a perforated tube, with some antiseptic fluid, particularly in blennorrhea of the sac and duct.

Argyll-Robertson said that he was personally cognizant of the employment of very large probes for eighteen years, which were introduced by Mr. Couper, of London, and he thought that such credit as attaches to this procedure should be given to him.

Theobold closed the discussion by saying that relapses undoubtedly occurred after treatment by his method, but such relapses were extremely rare. He thought their employment did not destroy the drainage-function of the passages.

Stoelting (44) performed repeated sclerotomies for hydrophthalmia, the patient being under chloroform anesthesia to prevent all sudden movement. The eyes are then bandaged closely, the first time for three days, the second for two days, and each time under chloroform. On the fifth day myotics and cold compresses are used. For each eye two sclerotomies were necessary. The process of cure lasted on an average fifty days.

Gutmann believed congenital hydrophthalmia is curable, if an operation is done early. He had found better results from iridectomy than sclerotomy.

Pflüger had been unfortunate in operating on many cases. The only case in which the cornea returned to its normal size and transparency, as well as function, was treated by sclerotomy, but as a routine method this operation cannot be recommended.

Thier thought good results could be obtained not only from iridectomy, but also from sclerotomy.

Dufour believed that, if the intra-ocular pressure could be reduced, even for a short time, the hydrophthalmia would be relieved. Myotics would sometimes do this; but if they failed, an operation would be necessary.

Malgat (45) had concluded that medical and hygienic treatment of conjunctival granulations were as imperative as surgical measures. For the surgical treatment he employed electrolysis, using a simple Gaiffe battery of seven pairs, provided with a rectilinear collector. The conductor carrying the needle is connected with the negative pole. With this every granulation (trachoma) is destroyed by applying the point of the needle successively to each one. Several sittings are required at intervals of two or three days. It is necessary to reverse the upper eyelid so as to expose the upper retrotarsal fold or resort to excision of it. The conjunctiva becomes smooth and normal without a trace of cicatrization. The electrolysis acts chemically, antiseptically, and as a means of modifying diseased tissues.

Chibret had investigated the etiology of trachoma for twenty years, and he believed that sun and air had nothing to do with its production, but that it was principally a disease of race, as had been shown by Swan Burnett, Hirschberg and himself.

Chavallereau (47) had treated two cases in whom paralysis of the oculo-motor nerve persisted eight months and six and onehalf months respectively, in spite of medical treatment. He cut the external rectus muscle in each case and the paralysis entirely disappeared.

MISCELLANEOUS PAPERS.

LEBER (23) recommended a new method of hardening and preserving eye-specimens by means of formol. The active principle of formol is formaldehyde, which is dissolved in water, to which is added a little methylic alcohol in the proportion of 40 to 100. To harden an eyeball the formol should be mixed with ten to thirty parts of water, the best proportion being about one to ten. Formol had been recommended the year before by Dr. F. Blum for the preservation of vegetable and animal substances and later by Dr. Edinger for the eyes.

Formol has no attraction for water, hardens tissues by coagulation and no shrinking takes place. It acts as a fixative for histological structures, requires a very short time (from one to a few days), the colors and even a part of the transparency of the media are preserved, the vitreus remains transparent and keeps its normal consistence and the common procedures of staining and embedding in celloidin, or paraffin, can be applied as well as after hardening in Müller's fluid, or by other methods, and no washing out of the hardening substance is necessary. After hardening, the specimens can be preserved in the same fluid or they may be mounted in glycerine jelly.

SWAN BURNETT (35) defended that system of measuring and designating prisms which made the prism-diopter (P. D.) or the centrad (Cr.) the standard or unit. He suggested as an exponent the sign △, instead of that of the degree (°).

SAVAGE (48) read a paper on the relation of oblique astigmatism to the oblique muscles of the eyes and gave practical demonstrations of the principles for which he contended. This subject is so new and its importance seems so great that quite a full abstract is hereby submitted.

The simple function of the oblique muscles is to counteract the torsion effect of the action of the superior and inferior recti and also of the external and internal recti, if by malposition these are inserted too high or too low. In other words, their function is to keep the normal vertical meridian of one eye parallel with that of the other in obedience to the law of corresponding retinal points. In oblique astigmatism the retinal images are distorted and in obedience to the same law, the oblique muscles are further brought into action to harmonize the retinal images. Thus, when the meridians of greatest curvature diverge above, the inferior obliques make the effort to harmonize the images, and when they converge above, the effort falls on the inferior obliques.

In applying a cylindrical glass for the correction of astigmatism, whether myopic or hypermetropic, there is in every case an arc of distortion for the superior obliques and also an arc of distortion for the inferior obliques. These two arcs always make 180 , but are not always equal. They are equal only when the axes of the cylinders are required at 90° or 180°. For plus cylinders the arc of distortion for the superior obliques is always wholly or in greater part in the lower temporal quadrant, while the arc of distortion for the inferior obliques is always wholly or in greater part in the lower nasal quadrant. The reverse is the case with the minus cylinders. The arc of distortion for the superior obliques is not always equal to the arc of distortion for the inferior obliques, but the one always supplements the other, so as to make 180°. To illustrate : If the arc of distortion for the superior obliques is 120°, that for the inferior is 60°. The point of maximum distortion in either of these arcs is just 45° from the vertical. The practical feature of this matter resides in the fact that we have within our power, by temporary rotation of cylinders, either plus or minus, in the proper arc from 5° to 10°, to relieve the patient of metamorphopsia and other uncomfortable symptoms which are so common in the great majority of cases of oblique astigmatism. Gradually these cylinders must be rotated back to the proper point, when the patient will remain comfortable. This is of special importance in cases where the obliques are insufficient. As the author of the paper has before stated, insufficiency of the superior obliques is a very common condition ; therefore, whenever oblique astigmatism is such as to call into activity the inferior obliques, it becomes a matter of very great importance in correcting this condition, to resort to the rotation of the cylinders so as to gradually transfer work from the inferior to the superior obliques. To illustrate : We have a case of hypermetropic astigmatism of 2°, the meridians of greatest curvature converging above. This uncorrected astigmatism distorts the images so as to throw a greater amount of work on the inferior oblique muscles. Correct this astigmatism by a plus cylinder, the distortion is at once reversed and the work to which the inferior obliques have been accustomed is at once transferred to the superior. The latter being weak, they do not take this extra work kindly. By revolving the axes of these cylinders towards 90° for each eye, through an arc of 5° or 10°, the images are distorted in the same way that the astigmatism itself distorts them, but objects are seen more sharply than if the lenses had not been given. This artificial distortion, while sharpening the images, leaves an excessive amount of work to be done by the inferior obliques. Gradually the cylinders are revolved back to their proper positions, and in this way, by small degrees, the work is transferred from the inferior obliques to the superior.

### INSTRUMENTS.

DARIER (13) exhibited aseptic collyria, an aseptic case for dressings, and a form of instrument for the shadow-test.

RISLEY (38) also showed an apparatus of his own construction for the shadow-test.

AYRES (39) presented a forceps for removing tarsal cysts and granulations of the conjunctiva.

### THE NEXT MEETING OF THE CONGRESS.

After the conclusion of the reading of the papers, the congress then took up for consideration the question of the place and time of the next meeting. Prof. Snellen, on behalf of his Dutch colleagues, invited the congress to hold its next session in Utrecht, Holland, and assured the members that they would do all in their power to make it interesting and profitable. He proposed that it be held in about five years hence, but at such a date that it would not clash with the meeting of the International Medical Congress.

Dr. Meyer, of Paris, said he had been commissioned by the Ophthalmological Society of Paris, and also by his French colleagues, to invite the congress to hold its next meeting in Paris in 1900, when the great international exhibition would be open in that city. Meetings of many other scientific bodies would be held at that time, and he thought it would be a good time and place for this congress. He could assure the congress of a most cordial and fraternal welcome.

Prof. Hansen-Grut, of Copenhagen, seconded the proposal of Dr. Snellen. He was afraid, if they went to Paris in 1900, there would be so many things to be seen outside that very little work would be done by the congress.

On putting the question to vote, the invitation and proposal of Prof. Snellen were accepted.

Prof. Snellen thanked the congress for this decision, and named as associated with himself on the committee of organization, Dr. Hansen-Grut, of Copenhagen ; Dr. Doyer, of Leyden ; Dr. Argyll-Robertson and Dr. Berry, of Edinburgh.

### CLOSING OF THE CONGRESS.

At this point Dr. Noyes, of New York, took the platform and, in a felicitous speech, proposed that hearty thanks be given to the official authorities of the University, and also to the College of Physicians and the College of Surgeons, for the complete, satisfactory and agreeable manner in which they had put at their disposal the facilities which the congress had enjoyed. He recalled a visit which he made to Edinburgh thirty years ago, and, noting the great expansion which the city had undergone in the interval, said he had been told that that expansion was due not to expansion in trade, in commerce, or in things of material character, but had been due to the expansion of learning. He commended the University for having chosen as the symbol which crowned the dome of its old buildings the figure of a youth looking forward to the career in front of him. That symbol represented an idea. It represented the beginnings of what they themselves were deeply concerned in. They were men of varied periods of life, but he was perfectly sure there was not one who had come to that meeting—coming as they had done from all parts of Europe and America—in whom did not burn the thirst for learning. In that institution they found a flame which should still further illuminate and warm their hearts ; and for all that those gentlemen had done in helping to carry forward successfully the meeting which now came to a close, he was certain that they would unite with him in rendering them most sincere and hearty thanks (applause). He begged to couple with his proposal a vote of thanks to the officers of the congress and to the members of the medical profession in the city (applause).

Sir William Muir, the Principal of the University, acknowledged the vote of thanks on behalf of the University, expressing his sense of the great service which the ophthalmological congress rendered, not only to medical science, but in the relief of humanity. Dr. Young, Treasurer of the Royal College of Physicians, and Dr. Littlejohn, President of the Royal College of Surgeons, returned thanks on behalf of their colleges, and expressed the pleasure they had experienced in entertaining the congress. The congress then rose.

(*Concluded next month.*)

